# Modelling the significance of organizational conditions on quiet quitting intention among Gen Z workforce in an emerging economy

**DOI:** 10.1038/s41598-023-42591-3

**Published:** 2023-09-18

**Authors:** Zhong Xueyun, Abdullah Al Mamun, Mohammad Masukujjaman, Muhammad Khalilur Rahman, Jingzu Gao, Qing Yang

**Affiliations:** 1 Faculty of Economics & Management, Universiti Kebangsaan Malaysia, 43600 UKM Bangi, Malaysia; 2 UKM - Graduate School of Business, Universiti Kebangsaan Malaysia, 43600 UKM Bangi, Selangor Darul Ehsan Malaysia; 3 Faculty of Entrepreneurship and Business, Universiti Malaysia Kelantan, Pengkalan Chepa, Malaysia; 4 Angkasa-UMK Research Academy (AURA), Universiti Malaysia Kelantan, Pengkalan Chepa, Malaysia

**Keywords:** Human behaviour, Psychology and behaviour

## Abstract

The phenomenon of “quiet quitting” has gained significant attention globally through various platforms, raising concerns about the impact of workplace stress on individuals’ personal lives and sparking social movements and investigations. As the number of Generation Z individuals is projected to surpass millennials by 2050, understanding and addressing the quiet quitting behaviour of this generation becomes crucial, considering their negative experiences during the COVID-19 pandemic and their preference for a work-life balance, which has led to a rejection of intense competition and a desire for a more relaxed lifestyle. Thus, this study investigated the factors (work conditions, job security, perceived career development opportunities, affective organizational commitment, and perceived organizational support on job burnout and employee well-being) determining the quiet quitting intention among Chinese Gen Z employees. It used an online survey to obtain cross-sectional data from 683 respondents, which were then tested using partial least squares structural equation modelling. The results showed that work conditions, job security, perceived career progression opportunities, affective organizational commitment, and perceived organizational support had a significant positive effect on employee well-being and that job burnout had a major negative effect. Furthermore, employee well-being had a significant negative impact on China’s Gen Z employees’ quit quiting decision and job burnout had a significant positive influence on China’s Gen Z employees’ quit quiting decision. The findings provide valuable insights for organizations and practitioners, enabling them to address these factors and effectively reduce quiet quitting intentions. Moreover, this study aligns with the Social Exchange Theory (SET), which explains how the interactions between employees and their organizations influence expectations and outcomes. By considering the SET framework, organizations can understand the motivations behind employees’ behaviours and make informed decisions to foster a positive work environment and enhance employee well-being.

## Introduction

The term “quiet quitting” has recently become a growing concern in communities worldwide via a range of platforms. It has become the focus of many social movements and investigations. The objective is to continue working rather than quit, so that the stressful events at the workplace are limited to working hours and do not affect one’s personal life. According to some reports, the term first emerged in 2009, and was suggested by an economist, Mark Boldger. This claim is corroborated by numerous websites^1^. The concept has recently become viral in China with the “Bailan (quiet quitting; lying flat; tangping or let it rot in English)” movement^2^, which is a sophisticated Chinese movement that resists a highly arduous and production-oriented society. A similar phenomenon was observed in the Gallup Report, which reported that the proportion of quiet quitters in the U.S. workforce is at least 50%, and in the second quarter of 2022, the ratio of engaged employees to actively disengaged employees was 1.8 to 1. Out of the total, 32% were engaged, while 18% were actively disengaged. According to Gallup’s longitudinal data, this marks the lowest level of engagement observed in the past decade^3,4^, a trend that has become increasingly widespread^2^. Zaid Khan’s (@zaidlepplin) TikTok video was particularly effective in spreading awareness on this issue. The TikTok user explains quiet quitting as follows: the employees are not quitting their jobs outright, but they are quitting the idea of going above and beyond the bounds of their work. The concept contends that quiet quitting is akin to the ancient union technique of working to dominate. This involves doing no more than what the contract requires. Unfortunately, this leaves little time for work’s increasingly valuable intangibles such as teamwork, idea-sharing, and creativity. Quiet quitting and “actual quitting” can be distinguished by the level of commitment and the overt expression of intentions to leave the organization. Quiet quitting refers to employees who demonstrate limited commitment to their assigned duties, often sticking to the minimum requirements of their job description^5^. They tend to be disengaged, lack initiative, and show reluctance to take on additional responsibilities^6^. Quiet quitters may gradually disengage without formally announcing their departure. On the other hand, “actual quitting” involves employees openly expressing their intention to leave through formal resignation processes or verbal communication^7^. They typically adhere to the organization’s notice period, engage in transition planning, and actively search for new job opportunities^8^. The distinction lies in the overt expression of departure intentions and the level of engagement and commitment exhibited by the employees.

By 2050, Gen Z (aged 18–28) is anticipated to surpass millennials in numbers, understanding and addressing the quiet quitting behaviour of Generation Z becomes imperative, considering that a significant portion of millennials are projected to retire by 2050. The quiet quitting phenomenon has had a stronger impact on Generation Z, as different generations have experienced the consequences of the COVID-19 pandemic in varied ways^9^. However, it is evident that Generation Z’s experience of the pandemic was overwhelmingly negative, primarily due to the challenging transition into adulthood, both personally and professionally^10^. This transition was fraught with difficulties and significantly affected their mental well-being. Notably, a substantial number of them encountered career disruptions due to widespread layoffs. Gen Z employees are not afraid to look for an organization that adheres to their work-life balance, which has led to a rejection of intense competition in favour of a more relaxed lifestyle^11^. The Gallup study^4^ found that 73% of American Gen Z employees left their jobs due to the mismatch with workplace expectations, and Deloitte^12^ reported that 61% of Gen Z employees plan to quit their jobs in the next two years. Thus, ensuring that the employees are invested in their work is essential to retain them in their jobs.

The “quiet quitting” in China has come to stand for the silent opposition of the populace to official ambitions. Approximately 68% of undergraduates stated in a survey by the China Youth Daily in 2021, that they expect to earn one million yuan ($155,000) per year within ten years of graduation. However, this time is characterized by owing to a record-high rate of youth unemployment (19.9%) and pricey housing^13^. At present, the sense of despondency among Chinese youth is further accentuated by dwindling economic opportunities as the world’s second-largest economy is struggling to increase growth^2^. China’s Gen Z, born and raised during the country’s heyday of economic expansion, face a constricted job market because of the economy’s worst growth in years^14^. Being meticulous is a sign of competence in Chinese culture. However, these factors have been combined to enrage the Chinese youth. A growing number of working-class people are compelled to work daily from 9 a.m. to 9 p.m., 6 days a week; yet they still cannot afford home loans or the cost of becoming parents^13^. Consequently, Chinese youth are protesting long office hours, wasteful spending, and rising housing expenses by working as little as possible^2^. This “quiet quitting” has come to represent the silent resistance of the population against official ambitions.

The existing academic literature has extensively examined factors influencing quitting intentions in various countries^15–18^. However, there is a lack of research specifically exploring the concept of quiet quitting intention among employees. Previous studies have highlighted the significance of job burnout in this context^19,20^. Additionally, there is a need for a comprehensive examination of the impact of work conditions^20^, job security^21,22^, perceived career development opportunities^23^, affective organizational commitment^24^, and perceived organizational support^25^ on the behavior of Generation Z employees across different work settings. The concept of quiet quitting is relatively new, resulting in limited scholarly research on the topic. While Zenger and Folkman^26^ were the first to uncover the link between bad bossing and quiet quitting in their article published in the Harvard Business Review, there is still much to be explored. Recent studies by Formica and Sfodera^3^, Scheyett^27^, and Salem^28^ have shed light on the problem of quiet quitting, but they have not delved into the underlying reasons behind employees choosing this form of resignation. Notably, limited empirical study has comprehensively investigated this phenomenon on a global scale or in the Chinese context. Employing social exchange theory (SET) as a lens for this study can provide insights into how the variables, including work conditions, job security, perceived career development opportunities, affective organizational commitment, and perceived organizational support, are interconnected. While these variables have been extensively explored in the literature on career management, the focus has predominantly been on pre-employment and educational factors, with limited discussion on the role of post-employment and management factors in facilitating young people's early career transitions. Therefore, this study aims to address this research gap and contribute to a deeper understanding of how employees perceive quiet quitting and identify the driving factors behind it, aiming to mitigate the potentially detrimental consequences of employee disengagement.

Investigative research has explored the mediating role of psychological contract violation between psychological contract breach factors and the related outcomes^29^, psychological contract breach and work-related behaviour^30^, and job insecurity, and intrinsic and extrinsic job satisfaction^31^. However, the study has not fully explored the moderating role of psychological contract violations. A few studies have examined the mediating effects of job burnout and employee well-being on turnover intention^32^, work misbehaviour^33^, productivity loss^34^, confidence in training^35^, employee engagement^36^, job performance^37,38^, and innovative work behaviour^39^. However, a mediating relationship between job burnout and employee well-being with the concept of “quiet quitting” has not been established. As this is a relatively new concept, it is important to investigate the mediator relationship between these variables to gain a better understanding of dormant and active turnover behaviour to address this gap.

This study aims to examine the effects of job conditions, job security, perceived career advancement opportunities, emotional organizational commitment, and perceived organizational support on employee well-being and job burnout, as well as the mediating effects of these factors on Gen Z’s intention to stay. For this, a comprehensive framework was developed and applied to Chinese Gen Z’s quiet quitting intention to fill the gaps in the existing literature. This research contributes to the academia and industry by providing the first empirical model to evaluate the global and Chinese quiet quitting intentions and by introducing new relationships to understand why employees quit without leaving physically. It helps organizations take proactive actions to reduce employee burnout and the intention to quit quietly. This study is structured to cover the literature and theoretical foundation (Sect. “Literature review”), methods (Sect. “Methodology”), results (Sect. “Results”), implications (Sect. “Implications’), and limitations and future research directions (Sect. “Conclusion’).

## Literature review

### Theoretical foundation

The social exchange theory (SET), formulated by Homans, Blau, and Emerson in 1958–1959, has been used to explain employee behaviour in the workplace and its connection with social capital research, particularly networks, norms, and trust^40^. Cropanzano and Mitchell^41^ explain that understanding subordinates in the workplace adds to the persuasive nature of the theory. Moreover, Jiwen et al.^42^ state that the idea of social exchange suggests that relationships between employees and the organization are exchanged, which can influence the organization’s performance.

SET is a theoretical framework that describes how employee perceptions of job scope and rewards interact with one another^43^. When an individual considers that the cost outweighs the benefit, the activity is avoided. If people do not value continuing or connecting with work, or if they are sceptical because they do not receive any reward, the balance changes toward expecting a lower value for any social deal^41^. Thus, elements such as work environment, job security, perceived career development opportunities, affective organizational commitment, and perceived organizational support, are founded on the SET framework, and they should be linked to job burnout, employee well-being, and a quiet intention to quit. SET can potentially explain how the appearance of perceived company support inspires better employee behaviour^40^. Owing to the difficulties in finding an acceptable theoretical model to comprehend quiet quitting intention, SET was chosen as an adequate theoretical model to analyse the quitting intention. A previous study also revealed that employee well-being may contribute to favourable behavioural outcomes such as lower turnover intention among employees^40^. Based on SET, the current study hypothesises that the effect of working conditions, job security, perceived career development opportunities, affective organizational commitment, and perceived organizational support on Gen Z’s quiet quitting intention is mediated by job burnout and employee well-being. As a result, this study creates a methodology for predicting quiet quitting intention among China’s Gen Z.

### Development of hypotheses

#### Work conditions, job burnout and employee well-being

Working conditions are implied to have two components in job analysis literature: dangers and environmental factors^44^. Environmental factors vary from common to extreme in terms of elements such as heat, humidity, sound, door, light, and dust. Harsh environmental circumstances have direct and indirect effects on employee performance. When exposed to such conditions, an employee’s ability to focus on duties declines, which leads to staff efficiency, low work quality, and emotional stress. Consequently, substantial costs are incurred. Risks, such as indirect or direct exposure to combustibles, light injury, burn danger, potential health risks from electrical devices, and deadly dangers, are often inevitable. Poor ergonomics are considered the primary contributors to workplace health risks and low levels of security^11^. The probability of accidents and injuries can be decreased by applying pertinent human-factor concepts. These effects lead to an increased absence from work and lower efficiency.

The literature on how work conditions can negatively impact mental and physical well-being is substantial^20^. It has been shown that inadequate workplace environments can lead to burnout, emotional stress, reduced employee satisfaction, physical complaints, and poor job performance^19^. This is probably a major contributor to high staff turnover, low employee satisfaction, and poor performance^20^. Workplace and employee happiness factors can be used to measure the efficacy of any changes or developments in the workplace. In addition, employees tend to perform better when the work environment is pleasant as it boosts their mood.

Recently, both practitioners and researchers have shown great interest in the well-being, a favourable psychological state that results from a person’s perspective, and the assessment of their life. Happiness and quality of life are two aspects of well-being; a multidimensional concept that involves physical, mental, social, and material well-being. Brunner et al.^45^ proposed the concept of “quality of life” as the state of the social and physical environment in which people fulfil their needs and desires. They also suggested that if employees are in unfavourable settings with high work demands, no value or respect, and lack of social support, their health and productivity will be affected through absenteeism and presenteeism. Using the social exchange approach, this behaviour can be seen as employees attempting to make the employer-employee relationship more equitable. Consequently, two hypotheses were formulated to test these theories.

- H_1A_: Work conditions negatively influence job burnout.
- H_1B_: Work conditions positively influence employee well-being.

#### Job security

Job security has recently been studied in both Western and Eastern countries. According to Altinay et al.^46^, it is a set of psychological states that employees experience in relation to their expectations of job stability in their organizations. Meret et al.^22^ conducted research in Italy and other European countries, which highlighted the importance of job security as a critical factor for Generation Z when selecting employment opportunities. The study emphasized the significance of job security in the decision-making process of this demographic. Furthermore, job security emerged as a primary concern for employees and exerted a more notable impact on their inclination to leave the organization, outweighing the likelihood of engaging in quiet quitting behaviour^47^. However, rapid organizational changes such as through outsourcing, mergers, and acquisitions, downsizing and restructuring can cause workers to feel increased job insecurity leading to powerlessness and burnout^46^. Turnover is just one of the many organizational behaviours triggered by anxieties, fear, and job insecurity brought on by ambiguous status, organizational or technical changes. Job insecurity and uncertainty disrupt career planning and other aspects of life^48^. Falatah et al.^21^ suggest that work security impacts more than an individual’s mental health, and that it also affects the whole family. To explore this further, the following two hypotheses were proposed.

- H_2A_: Job security negatively influences job burnout.
- H_2B_: Job security positively influences employee well-being.

#### Perceived career development opportunities

Perceived career development opportunities are defined as individuals’ chance to know themselves in the context of their job, and how to act on that information more consciously and creatively through a systematic, sequenced, and wide variety of experiences^49^. The process of designing a career path through education, training programs, and work experiences efficiently manages these plans^50^. Career development distinguishes itself from the conventional career approach by laying the groundwork for personal career advancement, while maintaining stability in the company and industry. Job satisfaction deteriorates without a professional development structure, while turnover increases. The positive impacts of well-directed personnel would make this method useful in a competitive setting over the long term despite the immediate costs it could incur for the firm^49^. A cross-sectional study conducted in China found that employees who perceive greater career development opportunities are less likely to experience burnout symptoms such as emotional exhaustion, depersonalization, and reduced personal accomplishment^51^. Burnout can be caused by job stress, which can make it difficult for individuals to fulfil their responsibilities in the workplace. Job satisfaction, career progression, and involvement in an organization have all been linked to decreased burnout^23^. According to Zacher and Rudolph^52^, when individuals are developing their careers, their job experiences help them develop their skills and learn about prospects both in the present and future. On the one hand, an employee may believe that there will be fewer options for them to work in the future. On the other hand, fulfilment is more likely to result in more optimistic ideas about future opportunities. The psychological contract of employees can inform them of the potential they have to reach their career goals, which can affect their job satisfaction and behaviour. Thus, the following hypotheses were proposed.

- H_3A_: Perceived career development opportunities negatively influence job burnout.
- H_3B_: Perceived career development opportunities positively influence employee well-being.

#### Affective organizational commitment

Meyer and Allen^53^ proposed that organizational commitment can be divided into three distinct categories: affective, continuance, and normative. Affective commitment or organizational commitment involves a deep emotional connection to identify with and participate in an organization. Affective commitment has stronger associations with positive outcomes, such as job satisfaction and lower turnover intentions, compared to the continuance and normative commitment. It reflects employees’ genuine emotional attachment to the organization. Affective commitment is closely related to employee well-being initiatives and work-life balance, and it plays a significant role in reducing burnout^54,55^. This study focuses on affective commitment to understand its impact on employee well-being, and engagement. An employee who feels obligated to stick to a firm in exchange for job stability is said to have a normative organizational commitment. Finally, continuing organizational commitment describes a worker’s need to remain with the company due to the perceived consequences of quitting. Koo et al.^56^ found that the relationship between burnout and organizational commitment is particularly significant because of the practical implications for organizations when a person has no other option or must give up too many benefits. Burnout also has significant dysfunctional effects that can have a notable financial impact on enterprises, including higher staff turnover, absenteeism, decreased productivity, and other personal issues. Some researchers such as Li et al.^25^, claimed that improved affective commitment and lowered turnover intentions among employees have both been connected to the availability of employee work-life well-being initiatives. The following hypotheses are based on this assertion.

- H_4A_: Affective organizational commitment negatively influences job burnout.
- H_4B_: Affective organizational commitment positively influences employee well-being.

#### Perceived organizational support

The level of support an organization provides to its employees reflects how the organization values their efforts and well-being^57^. When employees feel supported, understood, and their skills validated, it generates positive emotions and helps them recover from emotional fatigue caused by stressful work. Organizational support serves as an important external source of energy for employees, aiding in their emotional recovery during emotionally demanding tasks^58^. It signifies that organizations appreciate and care for their employees’ well-being, highlighting the strengths of the company^57^. This support includes assistance from managers, supervisors, and leaders in the workplace, and it stimulates high employee performance. Employees are drawn to organizations that fulfil their expectations in terms of their professional and personal ambitions^59^. Thus, we propose the following hypotheses.

- H_5A_: Perceived organizational support negatively influences job burnout.
- H_5B_: Perceived organizational support positively influences employee well-being.

#### Quiet quitting intention

Maryam et al.^15^ found that an employee’s intention to depart from an organization can be predicted by either their actual resignation or their intention to do so. Academics have considered job burnout as an explanatory mechanism for the associations between different stressors and turnover intention. Workplace pressures cause an adverse emotional reaction that results in job misery, which further motivates workers to retreat and disengage from their jobs to safeguard their psychological and emotional resources^60^. According to Aggarwal et al.^11^, work in organizations is evolving to be more complicated, dynamic, flexible, and virtual. To attract, inspire, and retain employees over the long term, there is a growing tendency to understand how human resource policies and practices affect job satisfaction. As employer branding and workplace connections are becoming important factors in organizational environments, profit development is no longer the main concern. Employers now give equal attention to employees’ well-being, work, and other factors. The “psychological contracts” idea, which has its roots in the SET, contends that principles of reciprocity and trade are at the core of employee-employer relationships^40^. Employees who feel deceived or their efforts are not acknowledged by the company may perceive this as a lack of gratitude for their accomplishments, which strengthens their desire to leave the company. In line with these arguments, we propose the following hypothesis.

- H_6A_: Job burnout positively affects the quiet quitting intention.
- H_6B_: Employee well-being negatively influences the quiet quitting intention.
- H_6C_: Psychological contract violation positively influences quiet quitting intention.

### Mediating and moderating effects

#### Moderating effect of psychological contract violation

Rousseau^61^ emphasized the significance of relational and transactional components in psychological contracts. The transactional aspect focuses on short-term, financial commitments, while relational contracts prioritize long-term, non-monetary interactions. The context influences the extent of resource loss resulting from a psychological contract violation^62^. Past experiences of psychological contract violations are considered important contextual factors. When employees perceive a violation of their psychological contract, the association between job burnout and their intention to quietly quit their job will be stronger^63^. According to Lin et al.^64^ and Morsch et al.^65^ the psychological contract serves as a mutual understanding between employees and their organization regarding expectations, obligations, and rewards.

Psychological contract violation and psychological contract breach are similar in that they both involve a perceived failure or discrepancy in the mutual expectations and obligations between employees and their organization^66^. While psychological contract violation focuses more on the employee’s subjective perception of unmet expectations, and psychological contract breach emphasizes the objective failure to fulfil obligations, they share the common ground of a breakdown in the employer-employee relationship and the resulting negative impact on employees^67^. According to Karani et al.^68^ found that psychological contract breach negatively impacted innovative behaviour and well-being, and job stress mediated the relationship between psychological contract breach and innovative behaviour as well as well-being during the COVID-19 pandemic situation. Studies such as Eckerd et al.^29^ have examined how psychological contract violation mediates the relationship between psychological contract breach factors and related outcomes.

Psychological contract violation and job burnout were specifically found to influence quiet quitting, which is a situation where employees mentally withdraw from their job but continue to physically work^69^. However, these factors may not have the same level of influence on actual quitting, which is when employees completely leave their job^70^. Employees can feel that their psychological contracts have been broken if their sense of well-being is not addressed by the employer. This is distinct from favours, which are reciprocal in nature, and there is evidence to suggest that when employee wellness is considered, withdrawal cognitions are reduced^71^. Consequently, we propose the following hypotheses.

- H_7A_: Psychological contract violation moderates the job burnout and quiet quitting intention.
- H_7B_: Psychological contract violation moderates the employee's well-being and quiet quitting intention.

#### Mediating effect of job burnout

Yang^72^ establishes that employee retention is significantly influenced by the workplace environment. This implies that employees who have a positive work environment are less likely to consider leaving the company. As employees face continuous stressors at work, they become more susceptible to experiencing burnout and it transfers the negative effects of work conditions on quiet quitting intention. As job burnout increases, employees may perceive quitting quietly as a way to escape the overwhelming stress and demands of their current work environment^73^. Similarly, when job security is at risk, employees may experience heightened anxiety and fear of job loss, leading to burnout and subsequent quiet quitting intention, as uncertainty about the organization’s stability can prompt them to leave without explicitly expressing their intention. This indicates that employees who perceive a lack of job security are more likely to experience burnout and develop quiet quitting intention^74^. Also, when employees perceive limited possibilities for advancement, they may feel stuck or stagnant in their roles, leading to feelings of disillusionment and burnout. Burnout, as a mediator, influences quiet quitting intention, as employees may decide to disengage quietly from their current position when they believe their career prospects are limited within the organization. As work conditions, job security, perceived career development opportunities, affective organizational commitment, and perceived organizational support is associated with reduced burnout and in turn a lower likelihood of quiet quitting^3,25,51^, we propose the following hypothesis.

- H_8A-E_: Job burnout mediates the effects of work conditions, job security, perceived career development opportunities, affective organizational commitment, and perceived organizational support on quiet quitting intention.

#### Mediating effect of employee well-being

Employees are more likely to feel positive emotions and be able to recuperate from the emotional exhaustion when they feel encouraged, and believe their skills are valued. Employee well-being is positively impacted by work conditions, job security, and organizational support, which acts as a crucial external source of energy for workers, helps them emotionally recover during emotionally taxing duties, and supports their emotional recuperation^11,22,57,58^. Scholars^3,75^ explored that perceived organisational support reduces quiet quitting intentions. Brunner et al.^45^ suggested seeking resources like favourable working conditions to achieve goals. Adverse work condition and perceived job insecurity can lead to heightened anxiety and negative emotions, thereby reducing overall well-being leading to the increased quiet quitting intentions. Zacher and Rudolph^52^ stated that employees stay when offered career development opportunities. Perceived career development opportunities are more likely to result in more optimistic ideas about future opportunities, therefore positively influence employee well-being^22,51^. Oyewobi et al.^24^ suggested individuals’ affective organizational commitment depends on perceived resources and demands. Reduced resources or excessive demands may threaten objectives and satisfaction, leading to career reconsideration. Organizational commitment, also known as affective commitment, requires a strong emotional bond in order to identify with and take part in an organization. Since affective commitment is more strongly associated with successful outcomes, it has a favorable impact on employee wellbeing^54,55^. Khan^59^ found corporate support aids stress management and work-life balance. Employee well-being, as a mediator, can transfer the positive or negative effects of these work-related factors on quiet quitting intention. When employees experience high levels of well-being due to supportive work conditions, job security, career development opportunities, affective organizational commitment, and perceived organizational support, they are less likely to consider quietly leaving their jobs. Conversely, the diminished well-being can lead to increased stress, emotional exhaustion, and a higher chance of considering quiet quitting as a way to escape the negative work environment. Consequently, we propose the following hypotheses.

- H_9A-E_: Employee well-being mediates the effects of work conditions, job security, perceived career development opportunities, affective organizational commitment, and perceived organizational support on quiet quitting intention.

## Methodology

### Data collection

An analysis of the correlations between variables was conducted using quantitative methods. A self-administered questionnaire was used for data collection, along with a cross-sectional research approach. This study examined the Chinese Gen Z, which constitutes 15% of the total Chinese population^76^. A sample size of 153 was determined using G ∗ Power 3.1, with an effect size of 0.15, a power of 0.95, and eight predictors^77^. Hair et al.^78^ suggested a sample size of at least 200 for partial least squares structural equation modelling (PLS-SEM). The 10 multiples rule suggested a sample size of 470^79^. A convenience sample was used to acquire an adequate number of respondents, and the questionnaire was shared on WeChat and Weibo applications. Informed consent was obtained, and 683 valid responses were collected over a period of four weeks in October 2022.

### Survey instrument

The questionnaire created for this study was composed of two parts: Sections A and B. Section A gathered demographic information, such as gender, age, monthly income, education level, and occupational status. Section B dealt with work conditions^19^, job security^80^, perceived career development opportunities^81^, affective organizational commitment^82^, perceived organizational support^83^, job burnout^84^, employee well-being^65^, psychological contract violation^63^, and quiet quitting intention^82,85^. From the previous studies, 47 items were adapted and rated on a seven-point Likert scale ranging from 1 (strongly disagree) to 7 (strongly agree).

### Common method bias

The collinearity of CMB was tested using the full collinearity test outlined by Kock^86^. As presented in Table 1, variance inflation factors (VIF) of less than five^87^ were found for latent constructs such as work conditions (1.553), job security (1.554), perceived career development opportunities (1.663), affective organizational commitment (1.585), perceived organizational support (1.852), job burnout (1.848), employee well-being (1.842), psychological contract violation (2.189), and quiet sitting intention (3.391). Subsequently, a single-factor test was conducted to assess the significance of the CMB. The results of the test showed that the common latent component accounted for 40.51% variation, which is insufficient for common method bias to be present^88^.

**Table 1:** Full collinearity test.

Variables	Variance inflation factors
Work conditions	1.553
Job security	1.554
Perceived career development opportunities	1.663
Affective organizational commitment	1.585
Perceived organizational support	1.852
Job burnout	1.848
Employee well-being	1.842
Psychological contract violation	2.189
Quiet quitting intention	3.391

Author’s data analysis.

### Multivariate normality

To select the optimal data analysis tool, the multivariate normality of the data was evaluated. Web Power online tool was used for the purpose and its results showed that the p-values for Mardia’s multivariate skewness and kurtosis were less than 0.05^89^. The analysis revealed a non-normality issue, leading to the use of PLS-SEM. (Source: https://webpower.psychstat.org/wiki/tools/index).

### Data analysis method

In this study, the two-step data analysis process of PLS-SEM with Smart PLS (V4.0) software was employed. This multivariate analytical tool enables the analyses of path models with latent constructs and non-normal data. Unlike covariance-based SEM, PLS-SEM can handle complex models with composites without requiring a goodness-of-fit assessment owing to its predictive nature^89^. In the first stage, the reliability and validity of the model constructs were evaluated^90^. The second step involved the assessment of the structural model and testing of any assumptions^89^. The results of (*r*^2^) and effect size (*f*^2^), which reflect the path impacts of exogenous constructions on endogenous constructs, were used for model estimations [902].

### Ethical statement

The human research ethics committee of Jishou University approved this study (Ref. Number: Jishou-2022-090101) This study has been performed in accordance with the Declaration of Helsinki.

### Informed consent

Written informed consent for participation was obtained from respondents who participated in the survey. No data was collected from anyone under 18 years old.

## Results

### Demographic details

This study included 683 respondents. Table 2 presents a demographic overview of the sample, of which 59.6% were male and 40.4% were female, and the largest age group was between 24–27 years old (43.9%), followed by 21–23 years old (36.6%), and 18–20 years old (19.5%). Most of the sample had a bachelor’s degree or equivalent (55.2%), followed by a diploma/technical school certificate (23.3%). Of the respondents, 52.9% were employed in the private sector, followed by public servants (31.2%), foreign companies (11.9%), and the self-employed (4.1%). Of the respondents, 80.7% were entry-level employees, 13% were intermediate or experienced (senior staff), and 3.5% were in first-level management roles. Executive or senior management was represented by 1% of the sample. Most respondents (70.9%) were single.

**Table 2:** Demographic characteristics.

	N	%
Gender		
Female	276	40.4
Male	407	59.6
Total	683	100.0
Age		
18–20 years	133	19.5
21–23 years	250	36.6
24–27 years	300	43.9
Total	683	100.0
Education		
Secondary school certificate	23	3.4
Diploma/technical school certificate	159	23.3
Bachelor’s degree or equivalent	377	55.2
Master’s degree	94	13.8
Doctoral degree	30	4.4
Total	683	100
Occupation		
Privately employed	361	52.9
Public servant	213	31.2
Foreign companies	81	11.9
Self-employed	28	4.1
Total	683	100
Working experience		
Less than 1 year	78	11.4
1 year to Less than 2 years	391	57.2
2–5 years	173	25.3
6–10 years	41	6.0
Total	683	100
Job level		
Executive or senior management	7	1.0
Middle management	12	1.8
First-level management	24	3.5
Intermediate or experienced	89	13
Entry-level	551	80.7
Total	683	100
Marital status		
Single	484	70.9
Married	159	23.3
Divorced	34	5
Widowed	6	0.9
Total	683	100
Average monthly income		
Less than CNY 1500	7	1.0
CNY 1500–CNY 3000	87	12.7
CNY 3001–CNY 4500	322	47.1
CNY 4501–CNY 6000	220	32.2
CNY 6001–CNY 7500	47	6.9
Total	683	100
Annual paid leave		
Less than 10 days	427	62.5
11–20 days	181	26.5
21–30 days	48	7.0
More than 30 days	27	4.9
Total	683	100
Average monthly absents		
0	525	76.9
1	122	17.9
2	24	3.5
3	6	0.9
4	5	0.7
8	1	0.1
Total	683	100

The survey results showed that most respondents (57.2%) had between one and two years of work experience. This was followed by the respondents who had two-five years of experience (25.3%), and the respondents who had less than one year of experience (11.4%). Only 6.0% of respondents had six-ten years of work experience. Most respondents’ average monthly income was CNY 3100–4500 (47.1%), followed by the groups earning CNY 4501–6000 (32.2%), CNY 1500–3000 (12.7%), CNY 6001–7500 (6.9%), and less than CNY 1500 (1.0%). Most respondents had less than 10 days of annual leave (62.5%), followed by the respondents who had 11–20 days of annual leave (26.5%), and the respondents who claimed that they had 21–30 days (7.0%) and more than 30 days (4.9%) of annual leave. Most respondents claimed that they had 0 average (monthly) absenteeism (76.9%) followed by those who claimed that they have 1 time (monthly) absenteeism (17.9%) (Table 2).

### Validity and reliability

To ensure the reliability of the questionnaire, Hair et al.^91^ suggested that Cronbach’s alpha and composite reliability’s rho should be used to determine internal consistency. The results in Table 3 reveal that all Cronbach’s alpha and composite reliability’s rho values exceeded the threshold value of 0.7, indicating the questionnaire’s reliability^92^. To ascertain convergent and discriminant validity, the average variance extracted (AVE) and factor loadings were used (Supplementary Material S1, Loading, cross-loading); the AVE values were greater than 0.5 for all latent variables^91^. Furthermore, the VIF values, which measure the level of collinearity in multiple linear regression models, ranged between 1.425 and 1.603, which did not exceed the threshold value of five recommended by Hair et al.^92^. The Fornell–Larcker criterion (See Table 4) values^93^ and the heterotrait–monotrait correlation ratio^94^ were also used to determine discriminant validity; both values were below 0.9, which empirically established effective distinctions (See Table 3).

**Table 3:** Reliability and validity.

Variables	Mean	Std. deviation	Cronbach’s alpha	Composite reliability (rho_a)	Composite reliability (rho_c)	Average variance extracted	Variance inflation factor
WCO	4.452	1.439	0.929	0.930	0.946	0.778	1.425
JSE	4.576	1.386	0.910	0.910	0.933	0.735	1.467
PCD	4.498	1.347	0.926	0.927	0.944	0.773	1.581
AOC	4.599	1.302	0.917	0.918	0.938	0.752	1.426
POS	4.643	1.306	0.926	0.927	0.942	0.730	1.569
JBU	3.326	1.405	0.935	0.936	0.949	0.756	1.549
EWE	4.821	1.329	0.915	0.916	0.937	0.747	1.588
PCV	3.157	1.424	0.939	0.940	0.954	0.805	1.603
QQI	3.151	1.433	0.909	0.910	0.933	0.736	

*WCO* work conditions, *JSE* job security, *PCD* perceived career development opportunities, *AOC* affective organizational commitment, *POS* perceived organizational support, *JBU* job burnout, *EWE* employee wellbeing, *PCV* psychological contract violation, *QQI* quiet quitting intention.

**Table 4:** Discriminant validity using Fornell-Larker and HTMT approach.

	WCO	JSE	PCD	AOC	POS	JBU	EWE	PCV	QQI
Fornell-Larcker criterion									
WCO	0.882								
JSE	0.421	0.857							
PCD	0.446	0.423	0.879						
AOC	0.323	0.414	0.452	0.867					
POS	0.435	0.446	0.484	0.436	0.854				
JBU	− 0.412	− 0.439	− 0.447	− 0.425	− 0.533	0.870			
EWE	0.482	0.426	0.441	0.475	0.526	− 0.473	0.864		
PCV	− 0.411	− 0.364	− 0.429	− 0.383	− 0.486	0.481	− 0.517	0.897	
QQI	− 0.504	− 0.512	− 0.552	– 0.531	− 0.594	0.634	− 0.570	0.737	0.858
Heterotrait–monotrait ratio (HTMT)									
WCO									
JSE	0.455								
PCD	0.480	0.461							
AOC	0.349	0.453	0.490						
POS	0.467	0.486	0.522	0.472					
JBU	0.439	0.476	0.480	0.458	0.572				
EWE	0.521	0.466	0.478	0.518	0.569	0.511			
PCV	0.438	0.393	0.459	0.412	0.520	0.513	0.557		
QQI	0.548	0.565	0.602	0.583	0.646	0.688	0.622	0.783	

*WCO* work conditions, *JSE* job security, *PCD* perceived career development opportunities, *AOC* affective organizational commitment, *POS* perceived organizational support, *JBU* job burnout, *EWE* employee wellbeing, *PCV* psychological contract violation, *QQI* quiet quitting intention.

### Hypothesis testing

**Figure 1:**
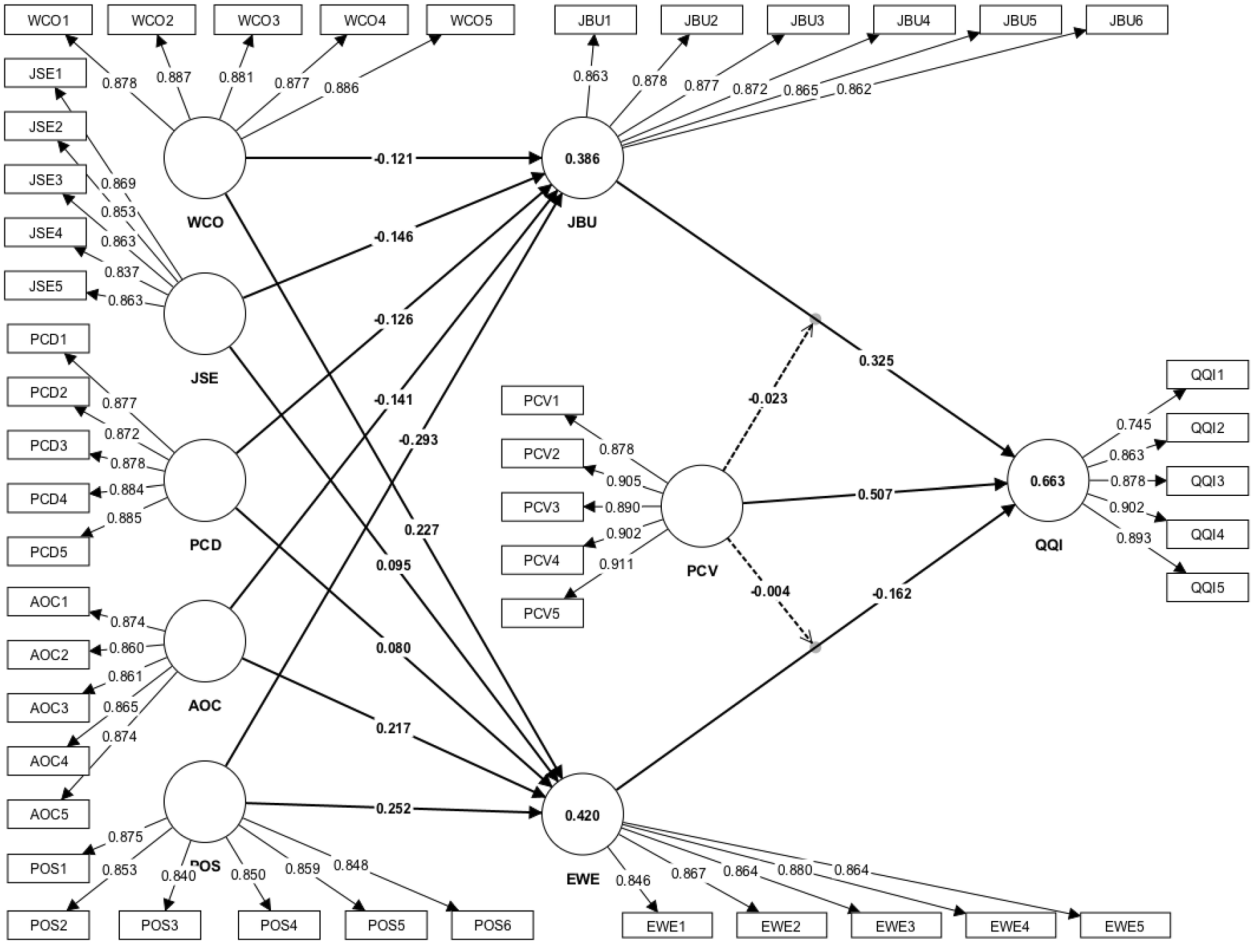
Measurement model.

This study evaluated the effect size (*f*^2^) and coefficient of determination (*r*^2^) values (presented in Table 5 and Fig. 1). The effect size results indicated that psychological contract violation had a substantial effect and job burnout had a considerable influence on quiet quitting intention, whereas the other effects were relatively minor^95^. The *r*^2^ value of 0.420 implied that 42% of the changes in employee well-being could be attributed to work conditions, job security, perceived career development opportunities, affective organizational commitment, and perceived organizational support^91^. Regarding job burnout, the *r*^2^ value of 0.386 suggested that 38% of the variation in job burnout could be attributed to the same factors. Finally, the *r*^2^ value of 0.663 showed that 66% of the changes in quite quitting intention were due to job burnout, employee well-being, and psychological contract violation.

**Table 5:** Effect size.

Variables	Effect size (*f*^2^)			Coefficient of determination (*r*^*2*^*)*
	EWE	JBU	QQI	
WCO	0.062	0.017		
JSE	0.011	0.024		
PCD	0.007	0.016		
AOC	0.057	0.023		
POS	0.069	0.089		
JBU			0.202	0.386
EWE			0.049	0.420
PCV			0.477	
PCV × EWE			0.000	
PCV × JBU			0.002	
QQI				0.663

*WCO* work conditions, *JSE* job security, *PCD* perceived career development opportunities, *AOC* affective organizational commitment, *POS* perceived organizational support, *JBU* job burnout, *EWE* employee wellbeing, *PCV* psychological contract violation, *QQI* quiet quitting intention.

*f*^*2*^ score interpretation (≥ 0.35- substantial effect size, ≥ 0.15– medium effect size, ≥ 0.02- small effect size and < 0.02- trivial effect size) (Cohen^95^).

As shown in Table 6, the path coefficients demonstrated the coefficient value for work conditions toward job burnout (β = − 0.121, t = 2.891, p = 0.002) which indicated that work conditions had a significantly negative effect on job burnout. The *f*^2^ value of 0.017 represents a small effect size^96^. The coefficient value for job security on job burnout (β = − 0.146, t = 3.418, p = 0.000) implied that job security had a significantly negative effect on job burnout. The *f*^*2*^ value of 0.024 denotes a small to medium effect size^96^. The coefficient value for perceived career development opportunities on job burnout (β = − 0.126, t = 2.765, p = 0.003) indicated that perceived career development opportunities significantly and negatively affected job burnout. The *f*^2^ value of 0.016 denotes a small media effect size^96^. The coefficient value for affective organizational commitment on job burnout (β = − 0.141, t = 2.981, p = 0.001) implied that affective organizational commitment had a significantly negative effect on job burnout. According to Cohen^96^, the *f*^2^ value of 0.023 suggests a small media effect size, while the coefficient value for perceived organizational support on job burnout (β = − 0.293, t = 6.088, p = 0.000) revealed that affective organizational commitment had a significant negative effect on job burnout. In comparison, the *f*^2^ value of 0.089 indicates a large effect size.

**Table 6:** Hypothesis testing.

Hypothesis	Associations	Beta	CI (Min)	CI (Max)	*T* value	*P* value	Decision
Factors affecting JBU							
H_1A_	WCO → JBU	− 0.121	− 0.190	− 0.051	2.891	0.002	Supported
H_2A_	JSE → JBU	− 0.146	− 0.216	− 0.076	3.418	0.000	Supported
H3_A_	PCD → JBU	− 0.126	− 0.202	− 0.051	2.765	0.003	Supported
H_4A_	AOC → JBU	− 0.141	− 0.219	− 0.063	2.981	0.001	Supported
H_5A_	POS → JBU	− 0.293	− 0.369	− 0.211	6.088	0.000	Supported
Factors affecting EWE							
H_1B_	WCO → EWE	0.227	0.161	0.291	5.625	0.000	Supported
H_2B_	JSE → EWE	0.095	0.023	0.165	2.187	0.014	Supported
H_3B_	PCD → EWE	0.080	0.014	0.151	1.916	0.028	Supported
H_4B_	AOC → EWE	0.217	0.146	0.290	4.943	0.000	Supported
H_5B_	POS → EWE	0.252	0.179	0.323	5.795	0.000	Supported
Factors affecting QQI							
H_6A_	JBU → QQI	0.325	0.260	0.384	8.755	0.000	Supported
H_6B_	EWE → QQI	− 0.162	− 0.217	− 0.109	4.931	0.000	Supported
H_6C_	PCV → QQI	0.507	0.452	0.559	15.577	0.000	Supported
Moderating effect of PCV							
H_7A_	PCV x JBU → QQI	− 0.023	− 0.067	0.023	0.840	0.201	No moderation
H_7B_	PCV x EWE → QQI	− 0.004	− 0.045	0.036	0.150	0.441	No moderation
Mediating effect of JBU							
H_8A_	WCO → JBU → QQI	− 0.039	− 0.064	− 0.016	2.708	0.003	Mediation
H_8B_	JSE → JBU → QQI	− 0.047	− 0.071	− 0.024	3.270	0.001	Mediation
H_8C_	PCD → JBU → QQI	− 0.041	− 0.068	− 0.016	2.561	0.005	Mediation
H_8D_	POS → JBU → QQI	− 0.095	− 0.127	− 0.064	4.895	0.000	Mediation
H_8E_	AOC → JBU → QQI	− 0.046	− 0.075	− 0.019	2.743	0.003	Mediation
Mediating effect of EWE							
H9_A_	WCO → EWE → QQI	− 0.037	− 0.055	− 0.021	3.589	0.000	Mediation
H_9B_	JSE → EWE → QQI	− 0.015	− 0.029	− 0.003	1.944	0.026	Mediation
H_9C_	PCD → EWE → QQI	− 0.013	− 0.027	− 0.002	1.708	0.044	Mediation
H_9D_	AOC → EWE → QQI	− 0.035	− 0.054	− 0.019	3.380	0.000	Mediation
H_9E_	POS → EWE → QQI	− 0.041	− 0.060	− 0.024	3.663	0.000	Mediation

*WCO* work conditions, *JSE* job security, *PCD* perceived career development opportunities, *AOC* affective organizational commitment, *POS* perceived organizational support, *JBU* job burnout, *EWE* employee wellbeing, *PCV* psychological contract violation, *QQI* quiet quitting intention.

The path coefficients demonstrated a coefficient value for work conditions toward employee well-being (β = 0.227, t = 5.625, p = 0.000) which indicated that work conditions had a significant positive impact on employee well-being. The *f*^2^ value of 0.062 denotes a large effect size and predictive relevance of work conditions toward employee well-being^96^. The coefficient value for job security on employee well-being (β = 0.095, t = 2.187, p = 0.014) indicated that job security has a significant and positive effect on employee well-being. The *f*^2^ value of 0.011 shows a small effect size^96^. The coefficient value for perceived career development opportunities on employee well-being (β = 0.080, t = 1.916, p = 0.028) implied that perceived career development opportunities have a significantly positive effect on employee well-being. The *f*^2^ value of 0.007 denotes a small media effect size^96^. The coefficient value for affective organizational commitment on employee well-being (β = 0.217, t = 4.943, p = 0.000) indicated that perceived career development opportunities had a significantly positive effect on employee well-being. The *f*^2^ value of 0.057 denotes a large effect size and the predictive relevance of employee engagement toward employee well-being^96^. The coefficient value for perceived organizational support on employee well-being (β = 0252, t = 5.795, p = 0.000) indicated a significantly positive effect on employee well-being. The *f*^2^ value of 0.069 denotes a large effect size and predictive relevance of perceived organizational support on employee well-being^96^.

### Mediating and moderating effects

This study analysed the mediating effects of employee burnout on the intention to quit and work conditions, job security, perceived career development opportunities, affective organizational commitment, and perceived organizational support in the Chinese Gen Z staff. It further revealed that job burnout had a significantly negative effect (p < 0.05) on the variables and the intention to quit. Similarly, employee well-being had a significant (p < 0.05) negative indirect effect on the same variables and quitting intention. The results are presented in Table 6. The findings revealed no moderating effect of psychological contract violation between job burnout and employee well-being on the quiet quitting intention. The path coefficients and f-values are presented in Table 6. Although the *f*^2^ value of psychological contract violation on quiet quitting intention is 0.477, indicating a large effect size of psychological contract violation on quiet quitting intention^96^,the *f*^2^ values of 0.000 and 0.002 denote the absence of an effect size of psychological contract violation toward employee well-being and job burnout on quiet quitting intention^96^.

## Discussion

This study examines the influence of various work conditions, such as job security, perceived career advancement opportunities, affective commitment to the organization, perceived organizational support, job burnout, employee well-being, and breach of the psychological contract, on the intention to quit without notice. Following a rigorous literature review, this study proposed a comprehensive research framework in which 25 hypotheses, including direct and indirect relationships, were proposed. The empirical investigation validated all the direct relationships (13) and 10 out of 12 indirect relationships. Thus, the proposed model fits well, as the explanatory power is strong (*r*^2^ = 0.663). The discussion of each relationship is as follows:

This study confirms that the work environment has a significant influence on employee well-being and job burnout (H_1A_ and H_1B_). This agrees with the previous study by Brunner et al.^45^, which showed that work conditions affect both employee well-being and burnout. Specifically, employees often experience high levels of burnout and emotional stress due to an unfavourable workplace environment. Next, the findings of one study indicated that job security has a negative impact on job burnout and a positive influence on employee well-being (H_2A_ and H_2B_). This result is consistent with the conclusions of Bušatlić et al.^48^. Discussions have revealed that Gen Z approaches their career differently compared to the earlier generations and they place importance on both financial stability and job security^97^.

In this study, perceived career development opportunities exhibited significant negative influences on job burnout and a positive influence on employee well-being (H_3A_ and H_3B_). These findings match the results of Kader et al.^23^, who postulated that career development opportunities correlated significantly with job burnout. This study corroborates the findings of Zacher and Rudolph^52^ that employees with a positive outlook on their future career prospects tend to be more productive and content with their jobs, and this has a beneficial effect on their well-being and behaviour. Furthermore, a recent study by Koo et al.^56^ found that organizational commitment is a key factor in job burnout. This finding is supported by the current study, which demonstrates that affective organizational commitment can have a beneficial impact on employee well-being as well as a negative impact on job burnout (H_4A_ and H_4B_). Oyewobi et al.^24^ also showed that affective commitment and lower turnover intentions among workers may be due to the availability of employee well-being practices.

Meanwhile, the findings of this study suggest that perceived organizational support has a negative effect on job burnout but a favourable effect on employee well-being (H_5A_ and H_5B_). This conclusion corresponds with the results of^57^, who showed that employees who experience bad treatment from their bosses, such as a lack of support and fulfilment, suffer from job burnout. Additionally, the findings confirm the assertions of Khan et al.^59^, who asserted that when organizations acknowledge their contributions, provide support, and offer a positive work environment, employees who are concerned about their well-being are more likely to stay with the organization. Subsequently, the findings of this study confirmed the hypothesis that job burnout and psychological contract violation have significant beneficial effects on the intention to resign (H_6A_ and H_6C_). This is in line with the findings by Park et al.^60^ and Karen et al.^40^ which indicate a positive relationship between job fatigue and psychological contract violation on quiet quitting intention. Additionally, Aggarwal et al.^11^ argued that job stress has a negative impact on employees’ personal lives, increasing their intention to leave. As employee well-being dampens quitting intentions (H_6B_), organizations that aim to create a supportive environment and improve employees’ perceptions of the job (e.g. positive attitudes regarding job responsibilities and work environment) are more likely to reduce turnover intention^62^. Human resource managers may also be able to reduce burnout and turnover intention by developing suitable treatments to deal with the proximal symptoms of psychological contract breach before they change to quiet quitting intention. Kurtessis et al.^58^ suggest that managers can modify the perception of job demands among Gen Z employees by providing training and organizational assistance. This could help employees meet job expectations and perceive lower job pressures, as their problem-solving abilities improve. Zhong et al.^62^ propose that organizations should promote strong bonds and care for formal and informal relationships to give employees a sense of belonging and being part of an extended family.

### Mediating and moderating effects

The study’s findings suggest that the interaction effect of psychological contract violation on the relationship between job burnout- quiet quitting intention and employee well-being- quiet quitting intention was not significant (H7A and H7B). This means that the degree of psychological contract violation did not significantly modify the association between job burnout/employee well-being and the intention to quit quietly. It is crucial to consider the role of psychological contract violation in influencing the impact of individual and contextual factors. Specifically, the inclusion of Generation Z individuals in the sample may explain the non-significant findings. Research has highlighted generational differences in work attitudes and organizational commitment perceptions. Generation Z employees, known for their shorter job tenures and higher inclination to explore various job opportunities, may not perceive psychological contract violation as a betrayal or experience the same level of emotional attachment to the organization as other generations. As a result, the emotional impact of contract violation on the relationship between job burnout and the desire to quit as well as well-being and the desire to quit may be less pronounced among generation Z employees. This suggests that the interaction effects between the breach of the psychological contract and job burnout, as well as employee well-being, may be attenuated in this generational cohort. Therefore, the moderating influence of contract violation on the main relationships may be influenced by the unique characteristics and perspectives of different generations.

Additionally, our findings confirm that job burnout mediates work conditions, job security, perceived career development opportunities, affective organizational commitment, and perceived organizational support on quiet quitting intention (H_8A_-H_8B_). These results suggest that when Gen Z employees perceive positive work conditions, job security, career development opportunities, organizational commitment, and support, it can potentially reduce the likelihood of experiencing job burnout. Therefore, organizations should prioritize creating a supportive work environment, providing career growth opportunities, and ensuring job security to mitigate job burnout and ultimately decrease the propensity for quiet quitting among Gen Z employees.

The findings of this study are supported by previous studies on the relationships between various work conditions and burnout symptoms. Wan et al.^73^ found that work conditions significantly contribute to the onset of burnout, and Bashir et al.^19^ found that job security also plays a role. Kader et al.^23^ found that perceived career development opportunities and Koo et al.^56^ found that affective organizational commitment are also linked to burnout. Kurtessis et al.^58^ found that perceived organizational support also contributes to burnout. Negative attitudes towards work, such as absenteeism and turnover, can have a negative impact on employee retention. These results suggest that employee retention is influenced by multiple factors, which collectively contribute to the overall outcome, including work conditions, job security, career development opportunities, affective organizational commitment, and perceived organizational support. To address these issues, the management should focus on improving work conditions, conflict management, communication, and provide more support to employees to mitigate the harmful effects of work patterns and arrangements. These interventions may help reduce the turnover rate and attract younger aspiring employees.

Finally, our data suggest that employee well-being serves as a mediator between work conditions, job security, perceived career advancement prospects, emotional organizational commitment, perceived organizational support, and the intention to quit one’s job. These findings support the idea that when employees experience favourable working conditions, such as the one listed above, they feel more content and experience a higher quality of life, which are both elements of well-being. This in turn increases their work engagement and performance, making them less likely to leave their jobs. This result is further supported by the results of prior studies by Brunner et al.^45^ on work conditions, Falatah et al.^21^ on job security, Zacher and Rudolph^52^ on perceived career development opportunities, Oyewobi et al.^24^ on affective organizational commitment, and Li et al.^25^ on perceived organizational support, which all demonstrate that negative attitudes towards one’s job can lead to an increased intention to quit, and that these factors significantly contribute to the development of employee well-being symptoms.

## Implications

This study makes several theoretical contributions to the relevant literature. First, this is the first empirical study to test quiet quitting intention based on employee perception. The previous studies on quiet quitting examined the preliminary aspects and were confined to conceptual development. The present study has come up with a detailed examination of organizational behaviour for the holistic understanding of quiet quitting behaviour. Second, this study seeks to add to the literature on organizational behaviour by taking a closer look at the world’s second-largest economy and the implications of quiet quitting intention on employee turnover, job satisfaction, motivation, and employee development. Third, the empirical validation of the proposed relationship in the Gen Z cohort will provide a comprehensive model for assessing quitting behaviour, and the scale generated can be replicated in various developed and underdeveloped economic perspectives. Fourth, the study offers new results and adds depth to the broader literature on violations. The previous study^29^ experimented with psychological contract violation as a mediating variable with various relationships, but they ignored its moderating roles. The present study empirically tested the relationship and found no relevant moderating variable within the job burnout—quiet quitting intention and employee wellbeing—quiet quitting intention relationships. although this relationship was not established in the current study, it paves the way for investigating other cohorts or other country settings using the established scale. Fifth, many studies have tested the mediating role of job burnout and employee well-being within various relationships with turnover intention, work misbehaviour, and so on. The current study, for the first time, tested the mediation relationship with the quiet quitting intention, which will be a point of reference for future work relevant to the human resource management literature.

The study also offers practical implications for the practitioners. First, since the study’s findings indicate that the work environment is significantly related to employee well-being and job burnout, organizations should take further action to enhance workplace conditions, such as implementing “open days” to familiarize new employees with their working environment. This approach helps alleviate concerns related to health and safety among Gen Z workers and equips them with the necessary resources to be effective in their roles. Onboarding practices like “open days” also reduce the uncertainty and anxiety commonly experienced by newcomers, improving their understanding of their responsibilities.

Second, Gen Z employees, who have experienced the Great Recession, prioritize factors like wages, benefits, job security, work-life balance, and flexibility. Unlike previous generations, Gen Z tends to get bored with routine tasks and values career growth opportunities. It is crucial for organizations to identify their skills and offer job rotation programs that allow them to collaborate with other departments aligned with their interests, promoting skill development. Providing “realistic job previews” that demonstrate potential career advancement can help applicants make informed decisions and set realistic expectations. Managers should encourage employees to discuss their future goals, provide realistic avenues for professional growth, and assign challenging tasks to foster opportunities for development^98^.

Third, the study emphasizes the importance of creating a supportive organizational culture that promotes employee welfare to ensure continued growth and dedication among personnel. Increasing emotional commitment and fostering a cooperative and empathetic work environment can reduce emotional weariness and ineptitude associated with job burnout, encouraging employee retention. To maximize the impact of organizational support, various human resource strategies should be adopted. These include implementing discretionary supportive workforce services, ensuring fairness and equity in management practices, setting achievable goals with appropriate rewards, offering individualized benefits, providing supportive training, and promoting strong social networks. Additionally, providing both material and non-material rewards directly reduce employees' intention to leave their jobs. Following the SET theory, when employees receive benefits that exceed their expectations, their employment becomes more meaningful. Thus, generating opportunities to improve employee well-being and welfare is critical. Managers should establish fair salary structures, and merit-based promotion criteria, and offer non-material advantages that align with employees’ abilities and tasks.

## Conclusion

This study aimed to comprehend the recent occurrence of quiet quitting intentions among the Chinese Gen Z. The evidence supports the notion that factors such as job environment, job stability, perceived chances for career advancement, emotional dedication to the company, and perceived company reinforcement significantly influence the Chinese Gen Z’s quiet quitting intention. This study created a comprehensive theoretical framework with eight intriguing components that influence Gen Z’s inclination to resign. This study also demonstrated that the social exchange theory is a good tool for estimating the probability of turnover among the Chinese Gen Z. In terms of practice, organizations should be encouraged to address the root causes of Gen Zs’ covert intentions to resign. For instance, emphasizing the importance of organizational commitment and support to develop a motivated and effective workforce. Additionally, the management needs to be aware that, over time, Gen Zs’ covert intent to leave can impair their performance.

Since the study had limitations, the findings are only suggestive rather than conclusive. First, the generalizability of the findings is limited by the small and specific sample size in the Chinese context. Also, it is often difficult to assess the representativeness of our obtained sample against the Generation Z population who are employed, as there are limitations in available statistics on this particular cohort in China.To gain a better understanding of this process in other contexts, it would be useful to replicate this study in other locations and sectors with a more representative sample based on the authentic population size.

Second, the cross-sectional design of the study did not allow for the determination of causal relationships between the variables. Future research using longitudinal data or experimental designs may provide further insight. Third, this study looks at employee well-being and job burnout as mediators, while future research may investigate other mediating factors such as work engagement and job satisfaction. To gain a more comprehensive and broader understanding of the factors contributing to quiet quitting, it would be useful for future studies to incorporate other constructs related to this phenomenon into the study model. This could provide a more nuanced and detailed understanding of the underlying causes and potential solutions to the issue of quiet quitting.

Fourth, this study did not find any moderating relationship psychological contract violation within the suggested relationships among Gen Z employees. Advanced studies in other settings can replicate the same investigation on other generations of employees, such as the millennials or older generations, to find its relevance in organizational behaviour research. Fifth, the limitation of this study is the potential for social desirability bias among participants. They may have been inclined to respond in a way that aligns with societal expectations and presents themselves in a positive light^99^. This could have influenced the self-reported data, potentially underestimating the prevalence of quiet quitting intentions. To address this limitation, future research could use alternative methods, such as qualitative interviews or observational studies, to gain a more comprehensive understanding of the factors influencing quiet quitting among Chinese Gen Z employees. Fifth, one limitation of this study is that the study did not consider demographic variables such as age, and gender as a control variable. Future research should aim to incorporate demographic variables as control factors to better understand their potential influence on the relationships under investigation. Another potential limitation in our study related to the possibility of endogeneity in affective organizational commitment and perceived organizational support. Although VIF analysis indicated no serious endogeneity problems with the exogenous variables, there is still a conceptual concern regarding the influence of other factors within the research framework. Future research could employ advanced statistical techniques or alternative research designs to address this limitation and provide a more robust examination of such relationships.

### Supplementary Information

Supplementary Information 1.

Supplementary Information 2.

## Data Availability

The original contributions presented in the study are included in the article/Supplementary Material, further inquiries can be directed to the corresponding author/s.
