# Supplementary material for: Modelling the significance of organizational conditions on quiet quitting intention among Gen Z workforce in an emerging economy
Source: Sci Rep. 2023 Sep 18;13:15438. doi: 10.1038/s41598-023-42591-3 (PMC10507021; doi:10.1038/s41598-023-42591-3)
Supplement: Supplementary file 1 — Supplementary Information 1. [file 41598_2023_42591_MOESM1_ESM.docx]

**Supporting Material 1.** Loading, cross-loading and Fornell-Larcker criterio

| Item Code | WCO | JSE | PCD | AOC | POS | JBU | EWE | PCV | QQI |
| --- | --- | --- | --- | --- | --- | --- | --- | --- | --- |
| WCO1 | 0.878 | 0.375 | 0.407 | 0.269 | 0.399 | -0.389 | 0.428 | -0.372 | -0.459 |
| WCO2 | 0.887 | 0.402 | 0.392 | 0.288 | 0.393 | -0.379 | 0.427 | -0.360 | -0.448 |
| WCO3 | 0.881 | 0.381 | 0.405 | 0.322 | 0.409 | -0.393 | 0.432 | -0.391 | -0.465 |
| WCO4 | 0.877 | 0.330 | 0.368 | 0.252 | 0.349 | -0.299 | 0.400 | -0.336 | -0.415 |
| WCO5 | 0.886 | 0.360 | 0.390 | 0.290 | 0.361 | -0.347 | 0.433 | -0.347 | -0.432 |
| JSE1 | 0.354 | 0.869 | 0.414 | 0.341 | 0.403 | -0.375 | 0.370 | -0.329 | -0.460 |
| JSE2 | 0.398 | 0.853 | 0.356 | 0.358 | 0.392 | -0.384 | 0.371 | -0.316 | -0.427 |
| JSE3 | 0.383 | 0.863 | 0.361 | 0.355 | 0.401 | -0.391 | 0.386 | -0.333 | -0.456 |
| JSE4 | 0.331 | 0.837 | 0.316 | 0.373 | 0.343 | -0.363 | 0.355 | -0.298 | -0.416 |
| JSE5 | 0.332 | 0.863 | 0.364 | 0.347 | 0.372 | -0.368 | 0.343 | -0.281 | -0.435 |
| PCD1 | 0.391 | 0.361 | 0.877 | 0.410 | 0.417 | -0.396 | 0.383 | -0.331 | -0.468 |
| PCD2 | 0.383 | 0.370 | 0.872 | 0.408 | 0.412 | -0.399 | 0.380 | -0.386 | -0.501 |
| PCD3 | 0.385 | 0.385 | 0.878 | 0.386 | 0.425 | -0.394 | 0.404 | -0.401 | -0.491 |
| PCD4 | 0.430 | 0.363 | 0.884 | 0.370 | 0.430 | -0.388 | 0.400 | -0.379 | -0.481 |
| PCD5 | 0.371 | 0.380 | 0.885 | 0.414 | 0.444 | -0.389 | 0.370 | -0.386 | -0.482 |
| AOC1 | 0.308 | 0.386 | 0.417 | 0.874 | 0.390 | -0.357 | 0.449 | -0.389 | -0.490 |
| AOC2 | 0.305 | 0.357 | 0.388 | 0.860 | 0.381 | -0.359 | 0.407 | -0.338 | -0.448 |
| AOC3 | 0.257 | 0.338 | 0.378 | 0.861 | 0.354 | -0.379 | 0.409 | -0.290 | -0.455 |
| AOC4 | 0.252 | 0.367 | 0.385 | 0.865 | 0.375 | -0.381 | 0.402 | -0.322 | -0.449 |
| AOC5 | 0.278 | 0.345 | 0.391 | 0.874 | 0.388 | -0.366 | 0.390 | -0.321 | -0.461 |
| POS1 | 0.400 | 0.385 | 0.423 | 0.409 | 0.875 | -0.493 | 0.468 | -0.454 | -0.544 |
| POS2 | 0.380 | 0.396 | 0.430 | 0.396 | 0.853 | -0.465 | 0.484 | -0.419 | -0.526 |
| POS3 | 0.370 | 0.388 | 0.402 | 0.342 | 0.840 | -0.435 | 0.440 | -0.371 | -0.467 |
| POS4 | 0.347 | 0.361 | 0.411 | 0.365 | 0.850 | -0.456 | 0.437 | -0.402 | -0.504 |
| POS5 | 0.384 | 0.370 | 0.422 | 0.340 | 0.859 | -0.459 | 0.431 | -0.442 | -0.496 |
| POS6 | 0.345 | 0.388 | 0.390 | 0.377 | 0.848 | -0.421 | 0.429 | -0.396 | -0.504 |
| JBU1 | -0.362 | -0.391 | -0.372 | -0.386 | -0.454 | 0.863 | -0.430 | 0.445 | 0.544 |
| JBU2 | -0.374 | -0.350 | -0.409 | -0.388 | -0.483 | 0.878 | -0.404 | 0.411 | 0.578 |
| JBU3 | -0.339 | -0.390 | -0.368 | -0.357 | -0.460 | 0.877 | -0.400 | 0.404 | 0.548 |
| JBU4 | -0.356 | -0.391 | -0.394 | -0.370 | -0.455 | 0.872 | -0.424 | 0.425 | 0.540 |
| JBU5 | -0.323 | -0.379 | -0.395 | -0.352 | -0.452 | 0.865 | -0.381 | 0.410 | 0.550 |
| JBU6 | -0.393 | -0.392 | -0.394 | -0.364 | -0.477 | 0.862 | -0.429 | 0.416 | 0.547 |
| EWE1 | 0.367 | 0.341 | 0.366 | 0.395 | 0.422 | -0.405 | 0.846 | -0.429 | -0.496 |
| EWE2 | 0.397 | 0.368 | 0.376 | 0.430 | 0.434 | -0.403 | 0.867 | -0.442 | -0.470 |
| EWE3 | 0.425 | 0.397 | 0.388 | 0.417 | 0.480 | -0.427 | 0.864 | -0.424 | -0.497 |
| EWE4 | 0.433 | 0.391 | 0.401 | 0.418 | 0.489 | -0.417 | 0.880 | -0.484 | -0.528 |
| EWE5 | 0.457 | 0.340 | 0.372 | 0.391 | 0.441 | -0.391 | 0.864 | -0.453 | -0.471 |
| PCV1 | -0.325 | -0.293 | -0.350 | -0.340 | -0.418 | 0.430 | -0.447 | 0.878 | 0.665 |
| PCV2 | -0.365 | -0.325 | -0.391 | -0.328 | -0.440 | 0.410 | -0.477 | 0.905 | 0.654 |
| PCV3 | -0.378 | -0.313 | -0.373 | -0.345 | -0.432 | 0.423 | -0.457 | 0.890 | 0.652 |
| PCV4 | -0.391 | -0.372 | -0.421 | -0.358 | -0.451 | 0.446 | -0.473 | 0.902 | 0.674 |
| PCV5 | -0.381 | -0.329 | -0.387 | -0.348 | -0.439 | 0.449 | -0.466 | 0.911 | 0.662 |
| QQI1 | -0.366 | -0.329 | -0.372 | -0.349 | -0.441 | 0.460 | -0.463 | 0.838 | 0.745 |
| QQI2 | -0.476 | -0.461 | -0.501 | -0.463 | -0.526 | 0.565 | -0.481 | 0.544 | 0.863 |
| QQI3 | -0.431 | -0.473 | -0.481 | -0.487 | -0.523 | 0.541 | -0.491 | 0.541 | 0.878 |
| QQI4 | -0.447 | -0.465 | -0.513 | -0.488 | -0.526 | 0.587 | -0.517 | 0.606 | 0.902 |
| QQI5 | -0.440 | -0.477 | -0.500 | -0.495 | -0.527 | 0.563 | -0.482 | 0.573 | 0.893 |

**Note.** Work Conditions (WCO), Job Security (JSE), Perceived Career Development Opportunities (PCD), Affective Organizational Commitment (AOC), Perceived Organizational Support (POS), Job Burnout (JBU), Employee Wellbeing (EWE), Psychological Contract violation (PCV), Quiet Quitting Intention (QQI)
